# Apigenin Alleviates Endotoxin-Induced Myocardial Toxicity by Modulating Inflammation, Oxidative Stress, and Autophagy

**DOI:** 10.1155/2017/2302896

**Published:** 2017-07-30

**Authors:** Fang Li, Fangfang Lang, Huilin Zhang, Liangdong Xu, Yidan Wang, Chunxiao Zhai, Enkui Hao

**Affiliations:** ^1^Department of Health, Jinan Central Hospital Affiliated to Shandong University, Jinan, China; ^2^Department of Obstetrics and Gynecology, Jinan Central Hospital Affiliated to Shandong University, Jinan, China; ^3^Central Laboratory, Jinan Central Hospital Affiliated to Shandong University, Jinan, China; ^4^Department of Cardiology, Qianfoshan Hospital Affiliated to Shandong University, Jinan, China

## Abstract

Apigenin, a component in daily diets, demonstrates antioxidant and anti-inflammatory properties. Here, we intended to explore the mechanism of apigenin-mediated endotoxin-induced myocardial injury and its role in the interplay among inflammation, oxidative stress, and autophagy. In our lipopolysaccharide- (LPS-) induced myocardial injury model, apigenin ameliorated cardiac injury (lactate dehydrogenase (LDH) and creatine kinase (CK)), cell death (TUNEL staining, DNA fragmentation, and PARP activity), and tissue damage (cardiac troponin I (cTnI) and cardiac myosin light chain-1 (cMLC1)) and improved cardiac function (ejection fraction (EF) and end diastolic left ventricular inner dimension (LVID)). Apigenin also alleviated endotoxin-induced myocardial injury by modulating oxidative stress (nitrotyrosine and protein carbonyl) and inflammatory cytokines (TNF-*α*, IL-1*β*, MIP-1*α*, and MIP-2) along with their master regulator NF*κ*B. Apigenin modulated redox homeostasis, and its anti-inflammatory role might be associated with its ability to control autophagy. Autophagy (determined by LAMP1, ATG5, and p62), its transcriptional regulator transcription factor EB (TFEB), and downstream target genes including vacuolar protein sorting-associated protein 11 (Vps11) and microtubule-associated proteins 1A/1B light chain 3B (Map1lc3) were modulated by apigenin. Thus, our study demonstrated that apigenin may lead to potential development of new target in sepsis treatment or other myocardial oxidative and/or inflammation-induced injuries.

## 1. Introduction

Sepsis affects significant number of people worldwide and has significant cost in emergency care and stunning high mortality rate [1]. Mortality rate is even higher in patients who have sepsis in past several years [2]. Cardiovascular risk is much higher among patients who have survived from severe sepsis [3]. Sepsis is attributed to a component of the cell wall of Gram negative bacteria: lipopolysaccharide (LPS). Cardiac dysfunction is one of the key factors in sepsis-related complications [4, 5]. The significant role of inflammation and associated activation of NF*κ*B in LPS-induced sepsis have been reported in the literature [6–8]. Oxidative stress in sepsis is well documented [9, 10]. The role of autophagy in sepsis is crucial but the relevant research is very limited [11, 12].

Incident of cardiovascular disease is rising due to epidemic of obesity, diabetes, and hypertension [13]. Increase of oxidative stress and chronic low-grade inflammation occurs in all these health disorders in addition to aging [14, 15]. Low-grade inflammation leads to circulating immune cells and platelets, which generate superoxide as byproduct of mitochondrial respiration or by enzymatic reactions such as lipoxygenases, myeloperoxidase, NADPH oxidases, and xanthine oxidase [16]. Thus, interplay of inflammation and oxidative stress plays critical role in cardiovascular disease.

Flavonoids, present in fruits and vegetables, are polyphenols and have great potential against cardiovascular complications including sepsis [17–19]. Apigenin, a flavonoid, is a traditional Chinese medicine isolated from celery *Apium graveolens*. Apigenin is also present in parsley and chamomile. Various researches have demonstrated that apigenin is anticancer, antioxidant, and anti-inflammatory [20–22]. Cardioprotective effects of apigenin have been reported in numerous studies. Apigenin ameliorates myocardial ischemia/reperfusion injury via the inactivation of p38 mitogen-activated protein kinase [23]. Apigenin reduces the blood pressure, heart weight, heart weight index, cardiomyocyte cross-sectional area, and serum angiotensin II in a cardiac hypertrophy model via HIF-1 and PPAR*α* pathways [24]. In an autoimmune myocarditis model of mice, apigenin mediates protection by inhibiting lymphocyte proliferation [25]. Apigenin attenuated proinflammatory cytokine expression by inactivating NF-kappaB through the suppression of p65 phosphorylation in vitro study of human monocytes and reduced LPS-induced mortality in mice [26]. In a LPS-induced endotoxemic rat model, apigenin attenuates heart injury by suppressing sphingosine kinase 1/sphingosine 1-phosphate signaling pathway [27]. We have shown earlier the role of autophagy, inflammation, and oxidative stress in sepsis model [28] and the cardioprotective role of resveratrol in LPS-induced myocardial toxicity via NRF2 [29].

In this study, we demonstrated that apigenin protected against LPS-induced cardiac tissue damage, cardiac injury, cardiomyocyte cell death, and cardiac dysfunction. Cardioprotection by apigenin was mediated by its anti-inflammatory and antioxidant effect. Autophagy by apigenin also played a role in cardioprotection.

## 2. Methods

### 2.1. Animal Experiments

Male C57BL/6 mice that are 4–6 weeks old were obtained from the Experimental Animal Center of Shandong University (Jinan, Shandong, China). LPS was purchased from Sigma (Beijing, China). LPS was dissolved in saline and administered intraperitoneally (i.p.) as described earlier [29]. The mice were given 4 mg/kg dose of LPS and kept for 18 hours for endpoint analyses. Apigenin (>98 purity) was purchased from Shanghai Winherb Medical S&T Development Co. Ltd. (China) and administered at 50 mg/kg of body weight intraperitoneally (i.p.) 1 hour post challenge of LPS. Vehicle for drug was 5% dimethyl sulfoxide (DMSO) in sterile saline. Mice experimental protocols were approved by the Institutional Animal Care and Use Committee of Shandong University and were in compliance with the Health Ministry of the People's Republic of China. Mice were sacrificed under deep anesthesia after completion of echocardiography.

### 2.2. Cardiac Injury and Tissue Damage Markers

Plasma CK and LDH levels were determined using an automated analyzer (Abbott Architect, Abbot Park, Illinois, USA) as described earlier [11]. Plasma cTnI concentrations were measured by ELISA-based assay according to the manufacturer's protocol (Abnova, Taiwan). Plasma cardiac myosin light chain-1 (cMLC1) was determined by ELISA (Life Diagnostics Inc., USA) according to the manufacturer's protocol. Both were described earlier [28].

### 2.3. Echocardiography

Echocardiographic cardiac parameters were determined as described earlier [29, 30].

### 2.4. Real-Time PCR

Total RNA was isolated by QIAzol method and reverse transcribed by OneStep Ahead RT-PCR Kit (Qiagen). All predesigned primers were purchased from Qiagen. mRNA level of TNF-*α* (tumor necrosis factor), IL-1*β* (interleukin 1 beta), MIP-2 (macrophage inflammatory protein-2), MCP1 (CD46), MAP1lc3 (microtubule-associated protein 1 light chain 3), VPS11 (vacuolar protein sorting-associated protein 11), or *β*-actin was detected by real-time PCR. The fold changes were determined by relative quantification method as described earlier [29].

### 2.5. Western Blot

Heart tissues were homogenized in lysis buffer, and protein concentration was determined as described earlier [29]. Nuclear and cytoplasmic fractions were isolated as described earlier [29]. PVDF membranes were incubated with TNF-*α* (1 : 200, Santa Cruz Biotechnology), MIP-2 (1 : 100, Abcam China), tubulin, NF*κ*B p65 antibody, anti-TFEB antibody, LAMP1, ATG5, p62, and histone H3 (1 : 200, Santa Cruz Biotechnology) overnight at 4°C. After three repeated washes, the membranes were probed with corresponding HRP-conjugated secondary antibody (1 : 2000, Rockland, Gilbertsville) for 1 h at room temperature. After three repeated washes, the membranes were detected by chemiluminescence and were exposed on an X-ray film for autoradiography.

### 2.6. Cardiac Cell Death Markers

DNA fragmentation was measured by ELISA-based kit (Roche) as described earlier [29]. For PARP activity, we used the HT Universal Colorimetric PARP assay kit from Trevigen as described earlier [31].

### 2.7. Cardiac TUNEL Staining

All TUNEL staining were performed with the In Situ Cell Death Detection Kit (Roche Applied Science) according to the manufacturer's instructions and published earlier [32].

### 2.8. Cardiac Glutathione Level

Cardiac glutathione levels from tissue lysates were determined by GSR-DTNB recycling assay and used in previous publication [33].

### 2.9. Cardiac Oxidative Stress Markers

Protein nitrotyrosine nitration was determined using OxiSelect™ Nitrotyrosine ELISA Kit (Cell Biolabs) as described earlier [12]. Carbonyl content in protein from tissue lysate was determined by Protein Carbonyl Colorimetric Assay Kit (Cayman Chemical) as described earlier [28].

### 2.10. Statistical Analysis

Data were expressed as mean ± standard deviation (SD), and statistical analysis was done by using GraphPad Prism software. Paired *t*-test or one-way analysis of variance followed by Tukey's posttest was performed and considered statistically significant.

## 3. Results and Discussion

### 3.1. Apigenin Attenuates Endotoxin-Induced Myocardial Injury and Cell Death of Cardiomyocytes in Mice

**Figure 1:**
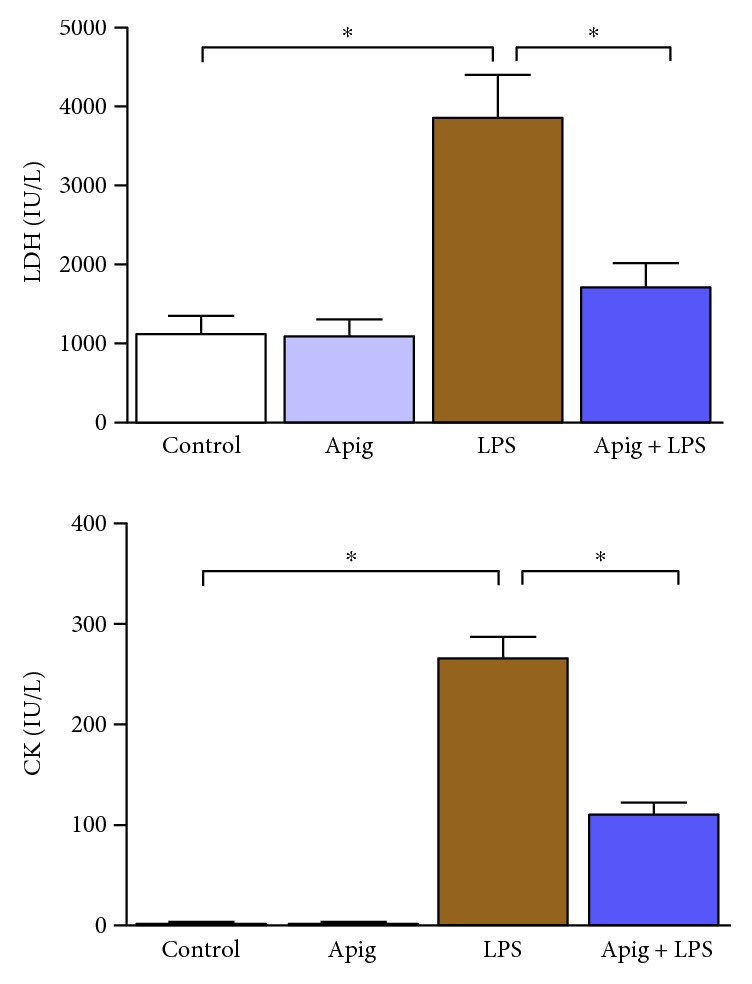
Apigenin attenuates LPS-induced cardiac injuries. Cardiac injuries were measured by plasma LDH and CK. LDH and CK plasma levels were increased in LPS-treated mice. Increase of LPS-induced cardiac injury was significantly attenuated by apigenin treatment. Values represented as means ± SD; ^∗^*P* < 0.05 and *n* = 6/group. Control was a vehicle-treated group where Apig was apigenin-treated group. LPS and Apig + LPS were administered with LPS along with posttreatment of vehicle or apigenin. Same nomenclature was used in all other figures.

**Figure 2:**
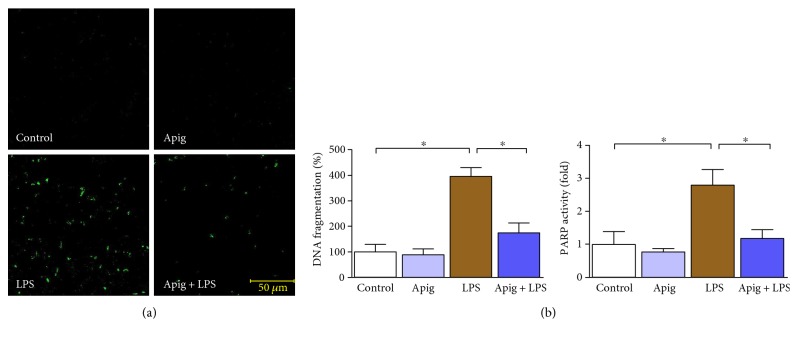
Apigenin attenuates LPS-induced cardiac cell death. (a) TUNEL staining performed on paraffin section of mice heart in each group and representative fluorescent images were provided. Green color demonstrated TUNEL-positive nuclei. Scale bar was provided in representative Apig + LPS image. (b) Cardiac cell death markers DNA fragmentation and PARP activity assay were examined. Both DNA fragmentation and PARP activity were significantly increased in LPS-treated mice, and apigenin treatment ameliorated those elevated level. Values represented as means ± SD; ^∗^*P* < 0.05 and *n* = 6/group.

**Figure 3:**
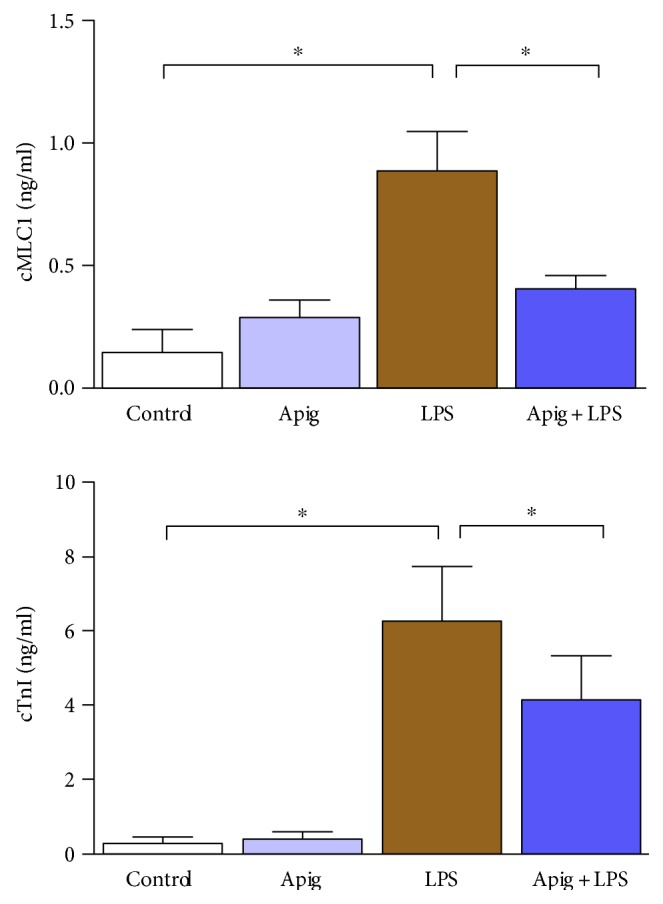
Apigenin attenuates LPS-induced cardiac damage. Cardiac damage was measured by plasma cTnI and cMLC1, which were secreted by damaged cardiomyocytes from the heart. Both of them were significantly increased in LPS-treated mice and were significantly attenuated by apigenin treatment. Values represented as means ± SD; ^∗^*P* < 0.05 and *n* = 6/group.

**Figure 4:**
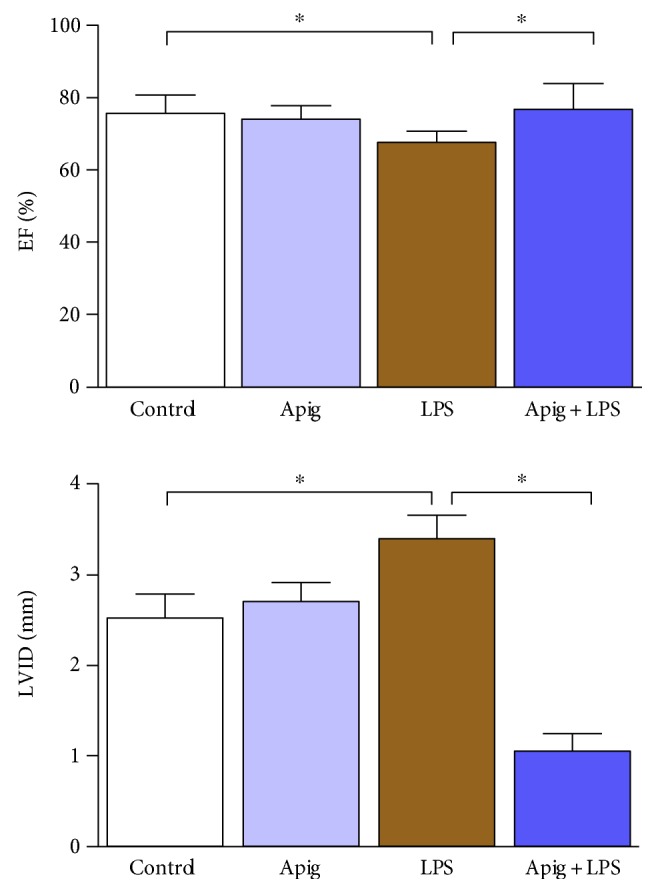
Apigenin improves LPS-induced cardiac dysfunction. Cardiac function parameters ejection fraction (EF) and left ventricular internal dimension (LVID) were measured by echocardiography. LVID was significantly increased whereas EF was decreased in LPS-treated mice. Apigenin reversed those changes and improved function. Values represented as means ± SD; ^∗^*P* < 0.05 and *n* = 6/group.

To examine cardioprotective effect of apigenin, we administered 4 mg/kg of LPS for 18 hours in C57BL/6 mice posttreated with either vehicle (control) or apigenin. LPS-induced myocardial injury was evident by increase in LDH and CK (Figure 1). Aginenin treatment significantly reduced LPS-induced myocardial injury. Cell death including apoptotic cell death markers were determined by TUNEL staining (Figure 2(a)). Quantitative cell death markers DNA fragmentation and PARP activity were determined and both were induced at 398% and 2.8-fold in response to LPS, respectively. Both markers, DNA fragmentation and PARP activity, were reduced to 55% and 57%, respectively, by apigenin treatment (Figure 2(b)). Cardiac troponin I (cTnI) and cardiac myosin light chain-1 (cMLC1) are proficient markers of acute heart disorders specifically for heart muscle cell death. cTnI and cMLC1 were induced by LPS administration to 6.3 ng/ml and 0.89 ng/ml in mice serum from 0.34 ng/ml and 0.14 ng/ml in the control group, respectively (Figure 3). Treatment with apigenin significantly attenuated LPS-induced serum cTnI and cMLC1 to 4.22 and 0.42, respectively. Cardiac function parameters such as left ventricular (LV) structure and function were assessed by echocardiography. LPS administration cause a decrease in ejection fraction (EF) and an increase of end diastolic left ventricular inner dimension (LVID), which was significantly attenuated by apigenin administration (Figure 4). The heart rates of the mice among all the four treatment groups were not statistically different. Treatment with apigenin alone did not alter any cardiac injury markers or function in the above experiments.

Multiple factors have been demonstrated to be involved in the endotoxin-mediated myocardial injury and cardiac dysfunction [5, 34–36]. Consistent with previous findings, our in vivo experiments indicated that LPS dramatically increased the plasma level of LDH, CK, cMLC1, cTnI, and cell death markers TUNEL staining, DNA fragmentation, and PARP activity. Apigenin treatment significantly reduced all the above markers. Primary mechanism of cell death in sepsis is by both apoptosis and necrosis with overlapping signaling pathways. In apoptosis, cell shrinkage and associated loss of myocardial structure leads to cardiac dysfunction [37]. In necrosis, an inflammatory response occurs, which also cause cardiac dysfunction [37]. We observed both types of cell death and apigenin-reduced cell death and cardiac dysfunction.

Pharmacokinetics study of apigenin in a rat model demonstrated that apigenin can be available in the system up to 10 days [38]. Apigenin can be found in human red blood cells after parsley consumption and thus increase its distribution and bioavailability [39]. We also observed that apigenin treatment improved LPS-induced cardiac dysfunction. In a long-term study of diabetic cardiomyopathy in mice, apigenin administration improves left ventricular functions in the heart [40]. Apigenin also improves the recovery of cardiac function during ischemia/reperfusion injury of isolated rat heart using Langedorff system [23]. Thus, our data on apigenin-mediated recovery of myocardial injury and cardiac dysfunction may lead to potential therapeutic development in sepsis.

### 3.2. Apigenin Suppresses Endotoxin-Induced Production of Proinflammatory Cytokines, NF*κ*B, and Oxidative Stress

**Figure 5:**
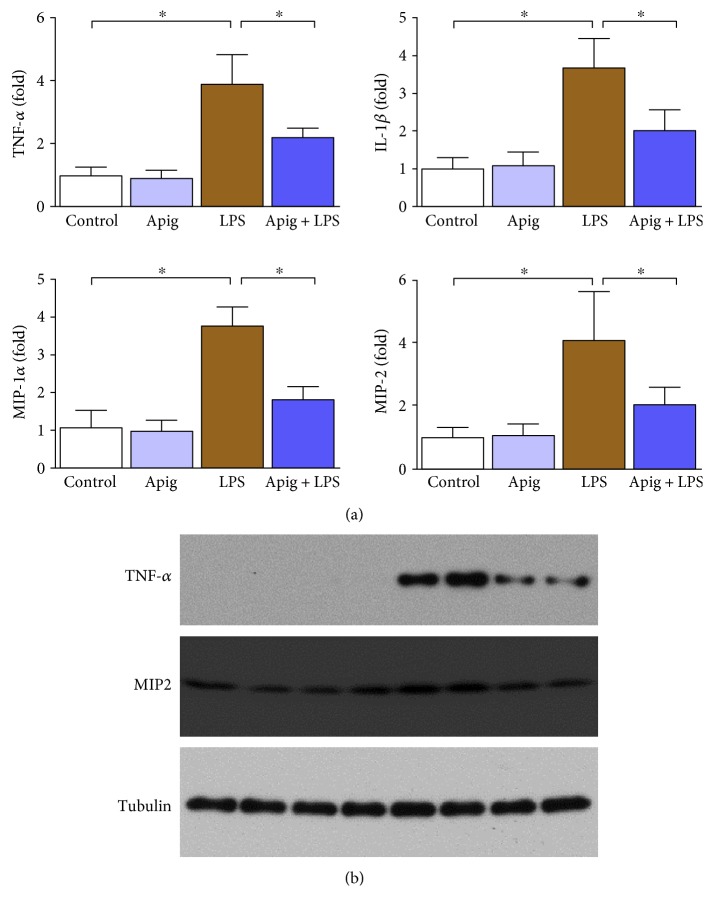
Apigenin attenuates LPS-induced cardiac proinflammatory cytokines. (a) Cardiac inflammation markers TNF-*α,* IL-1*β,* MIP-1𝛼, and MIP-2 were measured by real-time PCR, and apigenin treatment significantly attenuated LPS-induced proinflammatory cytokines. Values represented as means ± SD; ^∗^*P* < 0.05 and *n* = 6/group. (b) Protein level verification of TNF-*α* and MIP-2 performed by Western blot analyses where tubulin was used as a loading control.

**Figure 6:**
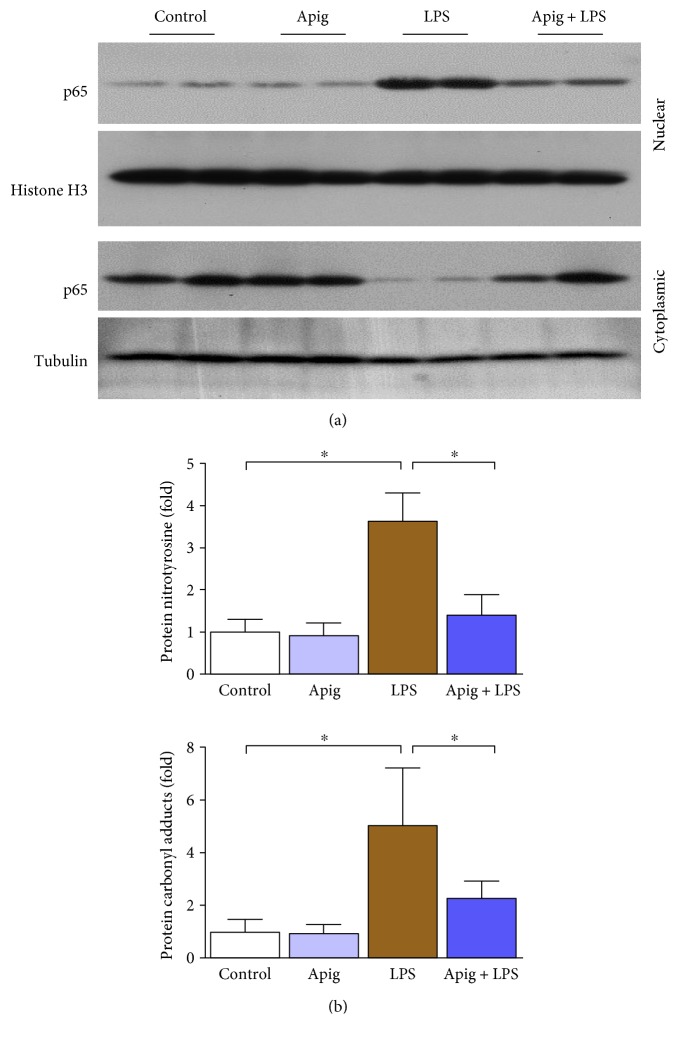
Apigenin attenuates LPS-induced cardiac NF*κ*B nuclear translocation and oxidative damage. (a) Western blot analyses of NF*κ*B (p65) in nuclear and cytoplasmic fractions from heart lysates. Histone H3 was used as a nuclear control whereas tubulin was used as a cytoplasmic control. (b) Cardiac oxidative markers protein nitration and carbonyl content measured by quantitative ELISA. All markers were significantly increased in LPS-treated mice. Apigenin significantly reduced LPS-induced oxidative stress markers. Values represented as means ± SD; ^∗^*P* < 0.05 and *n* = 6/group.

Real-time PCR analyses of proinflammatory cytokines demonstrated induction of TNF-*α*, IL-1*β*, MIP-1*α*, and MIP-2 by LPS administration up to 3.9, 3.7, 3.8, and 4.1, respectively. Apigenin treatment reduced LPS-induced cytokine gene expression by 43%, 45%, 51%, and 48% for TNF-*α*, IL-1*β*, MIP-1*α*, and MIP-2, respectively (Figure 5(a)). Two proinflammatory cytokines were verified at protein level, and it was consistent with mRNA level (Figure 5(b)). Western blot analyses demonstrated that NF*κ*B p65 was increased in nuclear fraction in heart lysate from LPS-treated mice and such increase was significantly reduced with apigenin treatment (Figure 6(a)). Similar analyses with cytoplasmic fraction also demonstrated that the cytoplasmic level of p65 was decreased by endotoxin and apigenin treatment restored to normal. NF*κ*B pathway is considered a conventional proinflammatory signaling pathway [41]. NF*κ*B is implicated in the expression of proinflammatory sources including cytokines, chemokines, and adhesion molecules. In sepsis, canonical pathways of NF*κ*B activation occurs [42]. Role of NF*κ*B activation is critical in inhibition of lipopolysaccharide-induced shock [43]. Thus, inhibition of p65 nuclear localization by apigenin in endotoxin-induced sepsis was beneficial.

**Figure 7:**
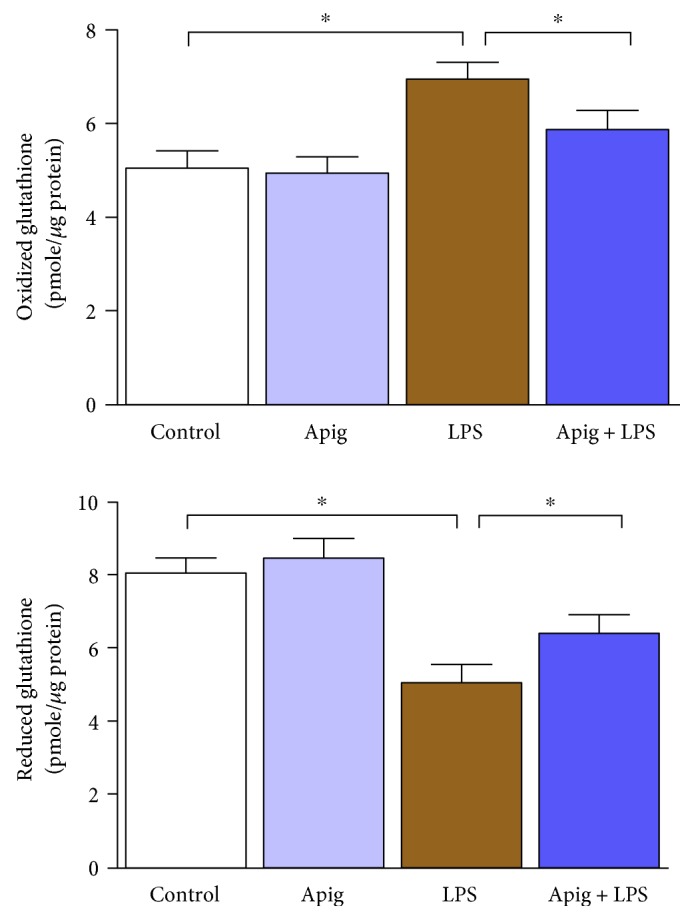
Apigenin restores LPS-induced redox status of glutathione. Apigenin restores LPS-induced oxidized glutathione. LPS downregulated reduced glutathione, which was restored by apigenin. Values represented as means ± SD; ^∗^*P* < 0.05 and *n* = 6/group.

Quantitative determination of oxidative stress markers protein nitrotyrosine and protein carbonyl by ELISA demonstrated significant increase 3.6 and 5.1, respectively. Apigenin attenuated LPS-induced protein nitrotyrosine and protein carbonyl by 61% and 515, respectively (Figure 6(b)). We also determined glutathione level in both oxidized (GSSG) and reduced (GSH) form. Endotoxin induced oxidized glutathione level and decreased reduced glutathione level, which were reversed by apigenin treatment (Figure 7).

Endotoxin-induced myocardial injury is associated with defects in redox balance and antioxidant enzymes, which leads to inflammation [44]. We have shown earlier the role of NRF2 in the sepsis and the protective effect by resveratrol [29]. In consistent with earlier literatures, we found increase in oxidative stress markers nitrotyrosine and protein carbonyl by LPS. LPS also induced inflammatory cytokines. Apigenin treatment significantly reduced both oxidative stress and inflammatory response. In a rat model of sepsis, cecal ligation and puncture induces oxidative stress and inflammatory response in the spleen and is attenuated by apigenin [45]. In another rat model of LPS-induced sepsis, apigenin ameliorates inflammatory response by suppressing sphingosine kinase 1 pathway, known as modulator of transcription factor NF*κ*B [27]. We also observed in this study that LPS-induced nuclear translocation of NF*κ*B was attenuated by apigenin. Apigenin inhibits the release of cytokines and suppresses NF*κ*B activity in LPS-stimulated monocytes isolated from human donor [26]. In a recent report, apigenin reduces NF*κ*B-associated neuroinflammation in a diet-induced obesity model of rat [46]. Thus, our study demonstrating apigenin as antioxidant and anti-inflammatory modulator via NF*κ*B in endotoxin-induced myocardial injury may be effective to other inflammatory heart injury models such as myocarditis.

### 3.3. Apigenin Modulates Autophagy in Endotoxin-Induced Cardiac Injury

**Figure 8:**
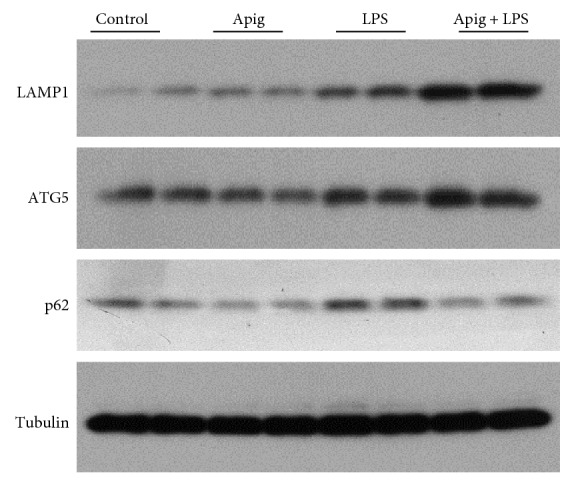
Effect of apigenin on autophagy pathway. Western blot analyses of three autophagy markers LAMP1, ATG5, and p62 along with control protein tubulin. Apigenin induces autophagy protein in LPS-treated mice where p62 is downregulated in the same samples.

**Figure 9:**
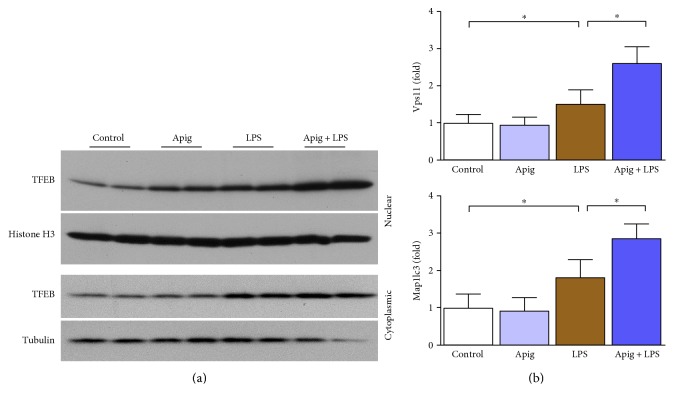
Apigenin enhances LPS-induced TFEB nuclear localization and its downstream genes Vps11 and Map1lc3. (a) Western blot analyses of TFEB in nuclear and cytoplasmic fraction from heart lysates. Histone H3 was used as a nuclear control whereas tubulin was used as a cytoplasmic control. (b) TFEB-regulated genes Map1lc3 and Vps11 were examined at mRNA level by real-time PCR. LPS induced mRNA level in both genes whereas apigenin further enhanced those mRNA expression. Values represented as means ± SD; ^∗^*P* < 0.05 and *n* = 6/group.

In animal model of sepsis and in clinical setting of sepsis, cardiac autophagy is increased [47]. Autophagy is also beneficial for cardioprotection during sepsis [48]. We examined autophagy by Western blot analyses of LAMP1, ATG5, and p62 (Figure 8). All three autophagy markers were increased in endotoxin-induced cardiac injury. Apigenin enhanced LAMP1 and ATG5 protein levels whereas p62 level was decreased by apigenin. These findings were in consistent with the fact that apigenin induced autophagy as p62 level was decreased and is a marker of autophagy induction in addition to be degraded by autophagy linking ubiquitinated proteins to the autophagic machinery in the lysosome [49]. Western blot analyses of nuclear fraction demonstrated that TFEB, a major modulator of autophagy and associated CLEAR pathway, was increased by both LPS and apigenin alone. Apigenin treatment along with LPS significantly enhanced nuclear localization (Figure 9(a)). To understand the functional role of nuclear translocation of TFEB, we determined two of its target genes Vps11 and Map1lc3 by real-time PCR (Figure 9(b)). LPS induced Vps11 and Map1lc3 gene expression by 1.53- and 1.83-fold, respectively. Apigenin treatment enhanced further LPS-induced gene expression to 2.6- and 2.9-fold for Vps11 and Map1lc3, respectively.

Flavonoids are known to modulate autophagy in a variety of pathological condition or physiological processes in animal models [50–53]. We have shown earlier that TFEB-mediated autophagy is modulated during aging [28]. In this study, we found that apigenin modulated autophagy pathway during sepsis. Curcumin, a flavonoid, also targets TFEB for induction of autophagy [54]. Recent literatures demonstrate the link of flavonoids with TFEB and/or autophagy [55–57]. Autophagy is also regulator for cardiovascular redox homeostasis and thus interplay of oxidative stress, autophagy, and inflammation is crucial in apigenin-mediated cardioprotection [58, 59]. We observed the protective role of TFEB and its downstream targets in LPS-induced myocardial injury and its modulation by apigenin. In doxorubicin-induced cardiomyopathy, TFEB is also modulated [60]. However, activation of autophagy has been identified as cardioprotective in some settings, but in other cases, sustained autophagy has been linked with cardiopathology [61]. Autophagy is thus known for its function as a double-edged sword [62, 63]. In this study, autophagy may work as redox homeostasis, enhancing the lysomal degradation of LPS-induced myocardial injury. Both are important for apigenin-mediated cardioprotection.

Our study on apigenin-mediated protection of endotoxin-induced myocardial injury demonstrated interplay of oxidative stress, inflammation, and autophagy. Increased cell death lead to increased inflammation, which is linked to increased oxidative stress. This process goes in cycle and thus enhances the damaging effect of endotoxin. Autophagy and antioxidant defense are also linked [64]. Autophagy regulates both redox balance and ROS formation, and autophagy plays a major role in the degradation of oxidized proteins [65].

## 4. Conclusion

**Figure 10:**
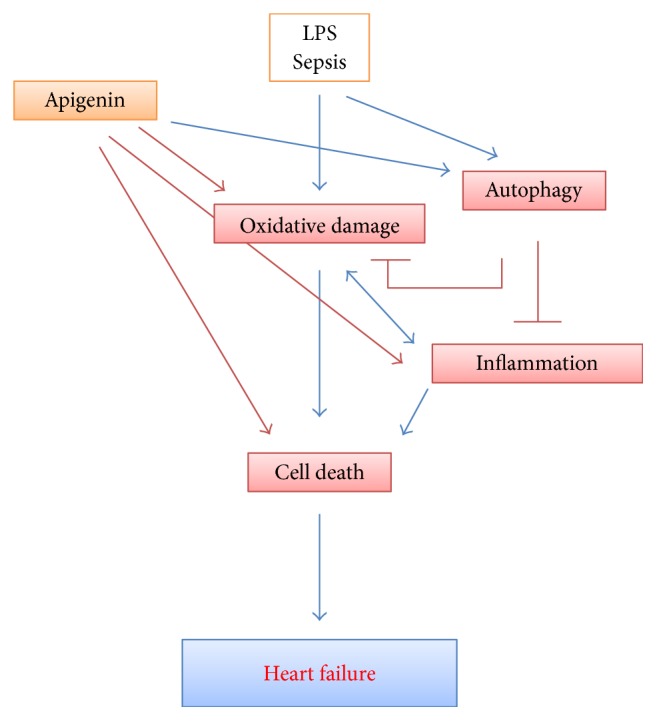
Schematic diagram of apigenin-mediated protection in LPS-induced myocardial toxicity and heart failure. Interplay of autophagy with oxidative damage and inflammation plays a critical role in pathophysiology of sepsis-induced cardiac dysfunction which results in heart failure. By enhancing autophagy, apigenin reduces oxidative damage, which also leads to inflammation and cell death.

We demonstrated that apigenin protects against LPS-induced cardiac injury, tissue damage, and cardiomyocyte cell death. Apigenin improves LPS-induced cardiac dysfunction. Apigenin-mediated protection against LPS-induced myocardial toxicity is mediated by multiple mechanisms where inflammation and oxidative stress were modulated by apigenin along with their master regulator NF*κ*B. In addition to that, autophagic pathway regulator TFEB was enhanced by apigenin. Due to autophagy enhancement, apigenin reduced LPS-induced inflammation and oxidative stress (Figure 10). Thus, the interplay of autophagy, inflammation, and oxidative stress and its modulation by apigenin played important role in cardioprotection.
